# Effectiveness of multimedia education for reducing anxiety among caregivers of children and adolescents undergoing chemotherapy: Randomized controlled trial protocol

**DOI:** 10.1371/journal.pone.0285250

**Published:** 2023-05-09

**Authors:** Silmara de Oliveira Silva, Fernando Hiago da Silva Duarte, Thatiane Monick de Souza Costa, Naryllenne Maciel de Araújo, Nahadja Tahaynara Barros Leal, Kleyton Santos Medeiros, Rodrigo Assis Neves Dantas, Daniele Vieira Dantas

**Affiliations:** 1 Nursing Graduate Program, Federal University of Rio Grande do Norte, Natal, Brazil; 2 Health Sciences Postgraduate Program, Federal University of Rio Grande do Norte (UFRN), Natal, RN, Brazil; 3 Instituto de Ensino, Pesquisa e Inovação, Liga Contra o Cancer, Natal, RN, Brazil; 4 Department of Nursing, Federal University of Rio Grande do Norte (UFRN), Natal, RN, Brazil; Brazilian national cancer institute, BRAZIL

## Abstract

**Introduction:**

Childhood cancer affects approximately 600,000 children and adolescents worldwide, and chemotherapy is the main form of treatment. However, chemotherapy treatment causes feelings of fear and anxiety especially in the patient’s caregiver. Thus, strategies that help the health education process directed towards caregivers are essential for strengthening knowledge and reducing anxiety involved with the beginning of treatment.

**Objective:**

To present a study protocol to evaluate the effect of a multimedia strategy compared to standard guidelines for acquiring knowledge and reducing anxiety among caregivers of children and adolescents with cancer undergoing chemotherapy.

**Methods:**

A randomized, controlled, single-blind, two-armed clinical trial will be carried out. Fifty-two caregivers of children and adolescents who will start chemotherapy will participate in the study, which will be randomly assigned into Experimental Group, which involves the evaluation of the effect of a multimedia strategy composed of a digital animation film about the chemotherapy process, used as tool for health education or into Control Group, which assesses the effects of standard guidelines, which are verbally provided. Two important moments will be considered to evaluate the results of the intervention (P1, and F1). The primary outcome includes reduced anxiety and the secondary outcome refers to the caregivers’ acquisition of knowledge about chemotherapy treatment.

**Expected results:**

The results of this randomized clinical trial will have a positive impact on the participants’ knowledge acquisition, and will also contribute to reduce anxiety observed at the beginning of treatment related to the caregivers’ knowledge deficit. The level of knowledge between groups with anxiety before and after intervention will be compared, highlighting which intervention had the best effect.

**Evaluation record:**

Registration: RBR-4wdm8q9–Brazilian Registry of Clinical Trials–REBEC (23/03/2022). This study was approved by the Research Ethics Committee of the Federal University of Rio Grande do Norte- UFRN, under CAAE—52597121.9.0000.5537.

## Introduction

Childhood cancer (from 0 to 19 years of age) represents a public health problem, affecting approximately 300,000 new children worldwide each year, with greater increase in developing countries. Furthermore, the World Health Organization (2022) estimates a prevalence of childhood cancer of approximately 600,000 cases worldwide, with leukemia, central nervous system tumors and non-Hodgkin’s lymphoma being pointed out as the most frequent [1].

It is noteworthy that childhood cancer presents a different behavior if compared to cancer in adults, being marked by uncertain mutations and non-evidenced risk factors, making it a challenge for the scientific community mainly related to early diagnosis and treatment [2, 3].

In this context, in relation to treatment, chemotherapy is the basis for therapy, and can be performed alone or associated with surgery and radiotherapy, considerably contributing to improve the prognosis and survival of patients [4].

However, chemotherapy treatment causes the development of negative feelings such as fear and anxiety among parents or caregivers, mainly related to the stigma of the disease, such as not knowing the therapy, its effects and the purpose of treatment [5].

Furthermore, it is in the impact of the diagnosis and definition of treatment that these feelings emerge, consisting a difficult period for the assimilation of information verbally transmitted by health professionals to caregivers. When information is transmitted in an accumulated way and with the use of technical language, caregivers /parents experience greater tension and lack of understanding about the disease and treatment [6, 7].

It is necessary to take into account that the caregiver may not have sufficient schooling to understand technical terms, and it is necessary to facilitate communication using language accessible to all, which should be enlightening, simple and objective [8].

Thus, the guidelines provided to caregivers / parents of children / adolescents undergoing chemotherapy are part of the activities of health professionals and among them the nurse, who plays an essential role in the health education process. Basic care with food, hygiene, hydration and management of chemotherapy side effects constitute important standard guidelines for caregivers and are provided by health professionals [9, 10].

In this context, the use of educational technologies that allow the insertion of resources that can contribute to the strengthening of health guidelines is highlighted. Therefore, the nurse, as an educator and care coordinator, must be on the developing team and use their knowledge and expertise of care in working with the team that develops these resources [11, 12].

Among tools included in the Educational Technology that can be used for the health education process are multimedia strategies, which represent a set of resources that stimulate all the senses, especially vision and hearing. Digital information can be transmitted through audio, video and animation simultaneously [13].

It is noteworthy that health education using multimedia before invasive procedures has shown significant results [14, 15], as an example, a randomized clinical trial that evaluated the effect of an educational video applied before the puncture procedure for intrathecal chemotherapy, demonstrated that the levels of palpitation, fear, dizziness and sweating were reduced in caregivers who watched the educational video before the procedure [8].

Thus, it is believed that the use of a multimedia strategy before chemotherapy administration can contribute to reducing the level of anxiety and prepare the caregiver for the management of children and adolescents undergoing chemotherapy. Based on the above, the study is justified due to the need to investigate the effectiveness of strategies aimed at supporting caregivers during cancer treatment. Furthermore, there are still few interventions using educational technologies for caregivers of children undergoing chemotherapy [16, 17].

Thus, the aim of this study was to evaluate the effect of a multimedia strategy compared to standard guidelines for acquiring knowledge and reducing the level of anxiety of caregivers of children and adolescents with cancer undergoing chemotherapy.

## Materials and methods

This protocol will adhere to the Consolidated Standards of Reporting Trials (CONSORT) [18] and Standard Protocol Items for Randomized Trials statements [19].

### Trial design

This is a protocol for a randomized, two-arm, controlled, interventional clinical trial with single-blind design that evaluated the effect of the multimedia strategy used as a tool for health education in the experimental group (EG) of caregivers, comparing it with the effects of instructions verbally, here called “standard guidelines”, provided to the control group (CG) of caregivers. Both interventions are related to children and adolescents with cancer undergoing chemotherapy treatment.

### Study scenario

Recruitment and intervention will be carried out at the largest philanthropic pediatric hospital that is a reference in the care of children and adolescents with cancer, located in the city of Natal, state of Rio Grande do Norte, Brazil.

### Study population

Caregivers of children and adolescents diagnosed with some type of cancer who will start chemotherapy treatment.

### Sample size

The sample is probabilistic and simple random. Sample size calculation was performed using the G Power software, version 3.1.9.2 (available at: http://www.gpower.hhu.de/). The calculation was performed considering the study by Bernardi et al. [20] that evaluated the IDATE score in caregivers of children and adolescents with cancer. Thus, considering the Cohen effect size of 0.80, test power of 0.80 and significance level of 5% (p-value < 0.05), the study had 26 participants in the Control Group (CG) and 26 participants in the Experimental Group (EG), totaling a sample of 52 study participants.

### Eligibility criteria

Caregivers of both sexes, aged 18 years or older, who are the main caregivers of the children or adolescent with cancer who will start treatment chemotherapy in the ward will be included.

Participants who present the following conditions will be excluded from the study: disorder that prevents understanding and participation in the research; caregivers of children and adolescents who are starting chemotherapy treatment with disease recurrence; caregivers who have already had experience in caring for patients undergoing chemotherapy, individuals with visual or hearing deficits; caregivers of children and adolescents undergoing outpatient chemotherapy.

### Recruitment

Caregivers will be recruited upon admission of the child/adolescent to the pediatric oncology sector of the hospital where the study will be carried out. Caregivers will be contacted and the eligibility criteria for participation in the research will be applied.

Subsequently, the study, interventions and instruments used for data collection will be presented to recruited participants and any doubts that may arise about the study will be addressed. Thus, participants who agree to participate in the study will receive a consent form for reading and signing. The study explanation and the free and informed consent form will be provided by the main researcher. It is noteworthy that participants will have their personal data protected.

### Allocation

The randomization of participants will be carried out through the www.randomizer.org website, which will previously select participants for each group (CG and EG) by simple randomness and without the influence of researchers. The site generates a list with the sequence of participants equally divided into the two intervention groups. It is noteworthy that the allocation of participants will be carried out by a researcher who is not involved in the intervention. Additionally, the allocation will be hidden.

It is noteworthy that randomization will avoid participant selection bias and will allow groups to be compared in terms of their known and unknown risk factors.

### Initial assessment

Participants who accept to participate in the research after signing the free and informed consent form will be presented to the study, interventions, instruments used for data collection and any doubts about the research will be cleared before intervention. After this step, participants will assigned one of two groups (CG and EG) according to the randomization list generated prior to the initial assessment.

### Intervention

The intervention that will be used in EG is the application of a multimedia strategy associated with the Institution’s standard guidelines. The multimedia strategy is the exhibition of a digital animation film with duration of 12 minutes and 22 seconds addressing the pediatric chemotherapy treatment process.

The film is an educational technology developed by Pinheiro et al. [21], during the master’s degree of the main author, using as reference a manual of a service of Hematology and Oncology of the University Hospital of Santa Maria (HUSM), specialized in the treatment of childhood cancer in partnership with students and professors of the Design, Digital Games and Journalism courses at the Franciscan University (UFN). It was validated by experts in the field and by the target audience and authorized to carry out the study, but it is not available for free access.

The digitally animated film will be shown in a quiet room, before starting chemotherapy. The caregiver will be provided with a headset and a tablet to watch the movie.

The digital animation film used in the study generally addresses the concept of chemotherapy, chemotherapy administration routes, most frequent side effects and general care during treatment. The film emphasizes health education about chemotherapy for the caregiver.

GC will receive instructions verbally. The standard verbal instructions are provided as supplementary material.

The intervention period will be carried out in two days consisting of three stages: the first stage is performed on the first day of contact after admission of the child/adolescent to the chemotherapy unit with the caregiver who, after accepting and signing the informed consent form, will be included in the search.

The second stage is performed on the second day of contact, which will be on the child’s first day of chemotherapy, and before starting the procedure, the main researcher will apply the instrument to assess the level of anxiety (State Trait Anxiety Inventory—STAI) of caregivers in both groups (CG and EG).

Subsequently, the intervention will be carried out according to the groups formed, in which the principal investigator will carry out health education using the multimedia strategy associated with the standard guidelines in the EG and standard guidelines in the CG.

The third stage will be carried out on the second day 30 minutes after the end of interventions in which a second researcher, trained to apply the STAI and KAI and who has no understanding of patient allocation, will apply these instruments to participants of both groups, CG and EG.

A pilot test will be carried out with the first nine participants, which will serve for research analysis in order to adapt instruments and the methodological approach as necessary to meet the objectives, for better adherence to the intervention protocol.

### Data collection instrument

Two instruments will be used for data collection:

1. State Trait Anxiety Inventory (STAI) [22], an instrument that assesses trait and state anxiety, the first relates to the caregiver’s usual anxiety and the second concerns transient anxiety related to some stressful event. Thus, the aim was to obtain the level of anxiety of participants before and after intervention, comparing these values to evaluate the management of anxiety by intervention, as well as comparing values between groups, evaluating the best management.
2. Knowledge Assessment Instrument (KAI), adapted from the study by Oliveira, Souza and Pellanda [23], which has the aim of assessing the effectiveness of the multimedia strategy used in the acquisition of knowledge by participants.

This instrument has a semi-structured questionnaire composed of three stages: the first consists of sociodemographic data of participants; the second contains information about previous guidelines by other professionals and the participant’s opinion about the intervention performed in order to report whether the intervention was able to resolve doubts about the chemotherapy procedure. The third stage consists of nine questions about the chemotherapy process, to quantify the knowledge that participants obtained on the subject after intervention. The STAI and KAI are validated and reliable instruments used in studies with adults and caregivers [22, 23].

The KAI and STAI instruments will be applied as an interview, clarifying the instrument’s questions and marking the alternative chosen by the research participant. It should be noted that STAI was translated and validated into Portuguese by Biaggio, Natalício and Spielberge [22].

The Knowledge Assessment Instrument (IAC) is a form prepared by the main researcher based on the questionnaire used in the study by Oliveira, Souza and Pellanda (2016) in portuguese.

**Fig 1:**
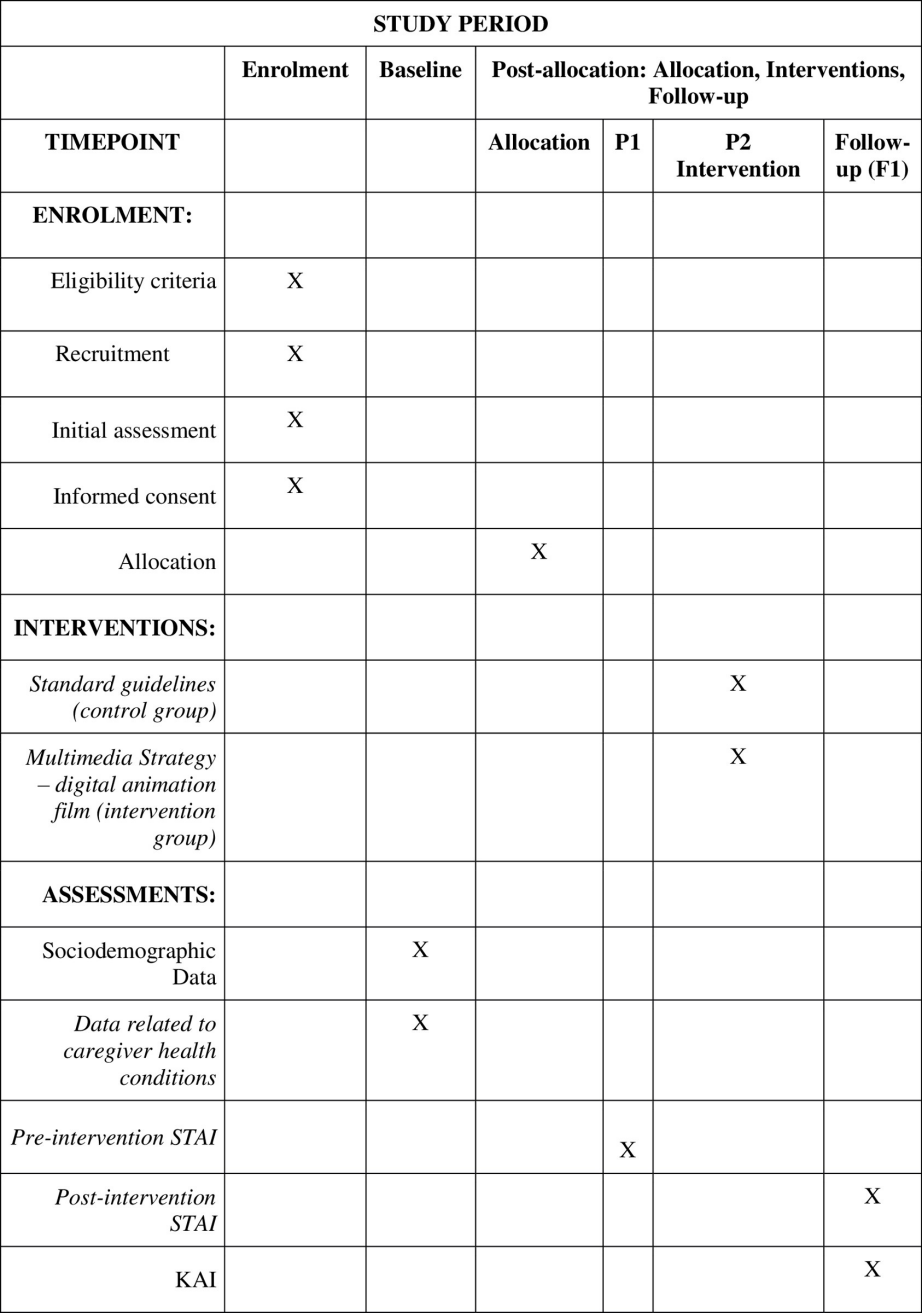
Description of the study stages. P1: Day 1—admission of the child/adolescent to start chemotherapy; **P2:** Intervention Period—(Day 2). **F1:** assessment 30 minutes after intervention. KAI: knowledge assessment instrument. **STAI:** State-Trait Anxiety Inventory.

Fig 1 shows the detailed steps of the study, with SPIRIT schedule of enrollment, interventions and assessments:

### Blinding

The second researcher responsible for the STAI and KAI application after intervention does not know the groups to which participants belong. In addition, data analysts will be blinded to the treatment group to which participants were randomized.

### Data collection and management

Researchers will ensure that the anonymity of participants is protected and data collected will remain confidential so that their identities and any type of identifying information are protected. Two copies of the informed consent form will be provided, both signed by participants, researchers and guardians.

After signing the consent form, the patient may terminate his/her participation in the study at any time, if desired, without any consequences related to his/her treatment or follow-up at the institution. The results of this research can be presented at meetings or publications; however, the identity of participants will not be revealed.

### Management of losses and dropouts

Losses and dropouts will be carefully recorded, ensuring research reliability. If any participant withdraws from participating in the study, it will be removed from the sample and another participant must be included. A logbook will be used to record information relevant to the study.

### Data analysis

Data collected in this research will be stored and processed in computerized database using the Microsoft Office Excel 2010 and Statistical Package for the Social Sciences (SPSS) version 20.0 software. Descriptive and inferential statistics of collected variables will be performed, and data will be presented through tables, charts and figures.

Statistical analysis of comparison and correlation of data obtained will be performed. The Kolmogorov-Smirnov test will be performed to analyze sample normality. Likewise, Pearson or Spearman tests will be used for correlational analyses. Categorical variables will be analyzed using the chi-square test or Fisher’s exact test. To compare the KAI and STAI assessment with the intervention and control groups, Student’s t-test will be applied. Comparing the IAC and IDATE classification with the group of intervention and control, the chi-square test (χ²) will be applied. Being adopted throughout the study a significance level of 5% at which p = 0.05.

### Ethical considerations

The present study was analyzed and approved by the Research Ethics Committee of the Federal University of Rio Grande do Norte–UFRN–CAAE—52597121.9.0000.5537 (03/02/2022)–and CEP 5.176.784 (03/02/2022). This study was also registered in the Brazilian Registry of Clinical Trials–REBEC–RBR-4wdm8q9 as a clinical trial (03/22/2022).

The participation of patients will be carried out on a voluntary, non-profit basis and all stages, objectives, risks and benefits of the research will be explained. Participants can drop out the study at any stage without prejudice to treatment, without suffering penalties or judgments of any kind.

### Primary outcomes

The primary outcome includes reduction in the level of anxiety of caregivers of children and adolescents undergoing chemotherapy. The before and after STAI anxiety data will provide the levels of anxiety of CG and EG to evaluate which intervention had the best effect.

Furthermore, data obtained on participants’ level of anxiety can be used to compare levels before and after intervention of each participant, whether in CG or EG; levels of anxiety between groups before and after intervention, assessing the management of anxiety according to the intervention performed.

STAI response possibilities range from 1 to 4, where: 1 = almost never; 2 = sometimes; 3 = often; and 4 = almost always. The final STAI score is directed to the level of anxiety classified as 20 to 40 points–low level; 41 to 60 points–moderate level; 61 to 80 points–high level.

### Secondary outcomes

Secondary outcomes refer to caregivers’ acquisition of knowledge about chemotherapy treatment. Thus, data will be generated on the level of knowledge of participants after intervention through the KAI, in which participants will have correct answers for questions scoring from zero (no correct answer) to nine points (all correct answers) and the knowledge acquisition can be analyzed in two ways: 1-KAI of verbal guidance participants (CG); 2- KAI of guidelines with educational technology (GE). KAI generates knowledge data on the subject after interventions, which can offer various combinations of analysis that aim to respond the research objectives.

In addition, it will provide data for the evaluation of each subject, offering an overview of the health education offered and which themes need greater intervention for a better understanding of caregivers. It is noteworthy that one of the aims of health education is to improve the knowledge of patients and caregivers, thus, knowing which topics are difficult to learn can provide evidence to improve guidelines [11].

## Discussion

Educational technologies are being gradually inserted in the context of pediatric oncology, requiring further discussion of the benefits of their use for caregivers of children with cancer undergoing chemotherapy. Resources that can help in the acquisition of knowledge in a playful way are essential for the effectiveness of cancer treatment [20].

In addition, one of the points that needs to be explored is related to the use of educational technologies that contribute to reducing the level of anxiety of caregivers of children / adolescents with cancer. It is observed in previous studies that videos and games have greater contribution to reduce the level of anxiety when compared with printed materials [8, 24].

A meta-analysis has shown that the prevalence of anxiety and depression in caregivers of cancer patients is approximately 47% and 42%, respectively, affecting their quality of life [25]. Corroborating this finding, the study by Medeiros et al. [26] stands out, in which the rate of common mental disorders (CMD), including non-psychotic depression, anxiety, and somatoform symptoms, was 93.1% in caregivers of children and adolescents undergoing chemotherapy and 96.5% of them reported feeling anxious/nervous or worried.

In this perspective, it was observed that physical symptoms resulting from stress and anxiety can be observed in caregivers of children undergoing chemotherapy, such as hypertension, headache, insomnia, increased body mass index (BMI) and significant immunological changes, such as increased concentration of cytokines and monocytes in the peripheral blood of these individuals [27].

These data are worrying, since increased level of anxiety is harmful to the caregiver’s quality of life as well as to the adaptation of children / adolescents to the chemotherapy treatment. It is necessary to investigate strategies that can contribute to improve caregivers’ quality of life, reducing the condition of mental disorders, anxiety and depression.

Thus, the development of studies with the purpose of investigating the benefits of using educational technologies to acquire knowledge and reduce anxiety in the context of pediatric oncology is essential, as both can influence the treatment of children with cancer.

Among the possible study limitations, it is highlighted that since it is a specific population, the sample may change due to the number of admissions during the collection period, making it necessary to recalculate the sample or extend collection.

### Dissemination of study results

The present study is part of an academic master’s degree. The dissertation and its results will be presented to the scientific committee of the Federal University of Rio Grande do Norte–UFRN.

### Study changes

Any necessary protocol changes will be effectively communicated and modified in the relevant parts (trial registry, funding agency and journal). Any questions about the study will be duly answered by the researchers in the initial evaluation period and during the study period.

### Study status

This study is currently being prepared for the recruitment of participants. It is scheduled to end in April 2023.

## Supporting information

S1 Checklist SPIRIT_Fillable-checklist-15-ago-2013. (DOC)

S1 Fig 2 CONSORT 2010 flow diagram. (TIF)

S1 File Default guidelines. (DOC)

S2 File Rebec. (PDF)

S3 File Ethics committee. (PDF)

S4 File Graphic outline for the protocol. (DOC)

S5 File Protocol committee. (DOC)

## References

[1] World Health Organization. Cancer incidence and mortality data: souces and methods by country [internet]. 2022. [cited 2022 abr 26]. Available from: https://gco.iarc.fr/today/data-sources-methods.

[2] Brasil. Instituto Nacional do Câncer. Estimativa 2020-Síntese de Resultados e Comentários. [internet]. 2020 [cited 2021 jul. 17]. Available from: https://www.inca.gov.br/estimativa/sintese-de-resultados-e-comentarios

[3] American Cancer Society. Risk factors and causes of childhood cancer. Atlanta: American Cancer Society [internet]. 2019 [cited 2021 jul. 24] Available from: https://www.cancer.org/cancer/cancerin-children/risk-factors-and-causes.html.

[4] Wakiuchi J, Marcon SS, Oliveira DC, Sales CA. Chemotherapy under the perspective of the person with cancer: a structural analysis. Texto Contexto Enferm. 2019;28(1): e20180025. Available from: 10.1590/1980-265x-tce-2018-0025

[5] Borrescio-Higa F, Valdés N. The Psychosocial Burden of Families with Childhood Blood Cancer. Int. J. Environ. Res. Public Health. 2022;19(1):599. Available from: doi: 10.3390/ijerph19010599 35010854 PMC8744617

[6] Mehdizadeh H, Asadi F, Mehrvar A, Nazemi E, Emami H. Smartphone apps to help children and adolescents with cancer and their families: a scoping review. Acta Oncol. 2019;58(7):1003–14. Available from: 10.1080/0284186X.2019.1588474 30915872

[7] El Muller, Cochrane AR, Campbell ME, Nikkhah S, Miller AD. An mHealth App to Support Caregivers in the Medical Management of Their Child With Cancer: co- designs and user testing study. JMIR Cancer. 2022;8(1): e33152 Available from: doi: 10.2196/33152 35293867 PMC8968552

[8] Hamdan AB, Ballourah W, Elghazaly A, Javison S, Alshammary S, Erlandez R, et al. The Effect of Video-Assisted Education Prior Intrathecal Chemotherapy on Anxiety and Knowledge Enhancemen. J Cancer Educ. 2020;20(1):65–70. Available from: 10.1007/s13187-020-01787-1 32519327

[9] Silva LN, Silva LF, Goes FGB, Machado MED, Paiva ED. Guidance on chemotherapy aimed at children with cancer: a sensitive creative method. Online Braz J Nurs. 2015;14(4):471–80. Available from: http://www.objnursing.uff.br/index.php/nursing/article/viewFile/5310/pdf_925.

[10] Sueiro IM, Silva LF, Goes FGB, Moraes JMRM. Nursing in Response to the Challenges Faced by the Family in Feeding Children in Chemotherapy. Aquichan. 2015;15(4):508–20. Available from: http://bases.bireme.br/cgi-bin/wxislind.exe/iah/online/?IsisScript=iah/iah.xis&src=google&base=LILACS&lang=p&nextAction=lnk&exprSearch=765441&indexSearch=ID. Acesso em: 08 jul. 2021.

[11] Kuntz SR, Gerhardt LM, Ferreira AM, Santos MT, Ludwig MCF, Wegner W. First transition from hospital care to home care for children with cancer: guidelines of the multiprofessional team. Esc Anna Nery. 2021;25(2):e20200239. Available from: https://www.scielo.br/j/ean/a/Z4RhCYkM69Tbw7v7GwkbKwj/?format=pdf&lang=pt

[12] Morrison CF, Szulczewski L, Strahlendorf LF, Lane JB, Mullins LL, Pai ALH. Designing Technology to Address Parent Uncertainty in Childhood Cancer. ANS Adv Nurs Sci. 2016;39(1):15–25. Available from: 10.1097/ANS.0000000000000100 26836990

[13] Sun V, Raz DJ, Ruel N, Chang W, Erhunmwunsee L, Reckamp K, et al. A Multimedia Self-Management Intervention to Prepare Cancer Patients and Family Caregivers for Lung Surgery and Post-Operative Recovery. Clinical Lung Cancer. 2017;18(3):151–159. Available from: https://www.ncbi.nlm.nih.gov/pmc/articles/PMC5413411. 10.1016/j.cllc.2017.01.010 PMC5413411 28233696

[14] Uzun Z, Kucuk S. Side effects of chemotherapy in children with cancer: effects of nursing training administered to caregivers. Aust J Adv Nurs. 2019;36(4):37–44. Available from https://www.ajan.com.au/archive/Vol36/Issue4/4Kucuk.pdf.

[15] Clercq E, Rost M, Gumy-Pause F, Diesch T, Espelli V, Elger BS. Moving Beyond the Friend-Foe Myth:A Scoping Review of the Use of Social Media in Adolescent and Young AdultOncology. J Adolesc Young Adult Oncol. 2020;9(5):561–71. Available from: 10.1089/jayao.2019.0168 32397793

[16] Mortola LA, Muniz RM, Cardoso DH, Azevedo NA, Viegas AC, Carniére CM. Educational video on oncological chemotherapy: technology in health education. Cienc Cuid Saude. 2021;20:e50365. Available from: 10.4025/cienccuidsaude.v20i0.50365

[17] Nova F, Allenidekania A, Agustini N. The effect of multimedia-based nutrition education on parents’ knowledge and body weight change in leukemia children. Enferm Clin. 2019;29(5):230–3. Available from: 10.1016/j.enfcli.2019.04.027

[18] Schulz KF, Altman DG, Moher D; CONSORT Group. CONSORT 2010 statement: updated guidelines for reporting parallel group randomised trials. BMJ. 2010;340:c332. Available from: https://bmcmedicine.biomedcentral.com/articles/10.1186/1741-7015-8-18 doi: 10.1136/bmj.c332 20332509 PMC2844940

[19] Chan AW, Tetzlaff JM, Gøtzsche PC, Altman DG, Mann H, Berlin JA, et al. SPIRIT 2013 explanation and elaboration: guidance for protocols of clinical trials. BMJ [internet]. 2013 [cited 2022 abr 25]; 8(1):346:e7586. doi: 10.1136/bmj.e7586 ; PMCID: PMC3541470. 23303884 PMC3541470

[20] Bernardi MLD, Amorim MHC, Salaroli LB, Zandonade E. Effects of Hatha Yoga on caregivers of children and adolescents with cancer: a randomized controlled trial. Escola Anna Nery. 2019; 24(1):e2019013. 10.1590/2177-9465-EAN-2019-0133

[21] Pinheiro M, Vieira AS, Sasso T, Oliveira MF, Abaid JLW, Filippin NT. We are your friends: a film of digital animation for children in chemotherapy treatment. Research, Society and Development. 2020; 9(12):2–15. Available from: 10.33448/rsd-v9i12.11253

[22] Biaggio AMB, Natalício L, Spielberger CD. Development of the experimental form in Portuguese of the State-Trait Anxiety Inventory (STAI): by Spielberger. Arquivos Brasileiro de Psicologia AplicadA. 1977; 3 (29): 31–44. Available from: http://bibliotecadigital.fgv.br/ojs/index.php/abpa/article/view/17827.

[23] Oliveira APA, Souza EM, Pellanda LC. Effectiveness of video resources in nursing orientation before cardiac heart surgery. Rev. Assoc. Med. Bras. 2016;62 (8):762–767. Available from: https://www.scielo.br/j/ramb/a/ydPt75qjBn4ghypvB5STx3G/abstract/?lang=en. doi: 10.1590/1806-9282.62.08.762 27992017

[24] Maza V, Fernández M, Concha L, Santolaya ME, Villarroel C, Castro M, et al. Impacto de un programa educativo a los padres de niños con cáncer en el aumento del conocimiento de la enfermedad de sus hijos y la disminución de la ansiedad. Rev Chil Pediatr. 2015;86(5):351–356. Available from: 10.1016/j.rchipe.2015.04.027 26593888

[25] Geng HM, Chuang DM, Yang F, Yang Y, Liu WM, Liu LH, et al. Prevalence and determinants of depression in caregivers of cancer patients: A systematic review and meta-analysis. Medicine (Baltimore). 2018;97(39):e11863. doi: 10.1097/MD.0000000000011863 ; PMCID: PMC6181540. 30278483 PMC6181540

[26] Medeiros JRA de, Carvalho MAP de, Medeiros APG, et al. Common mental disorder among caregivers of children. Rev enferm UFPE. 2018;12(3):651–7. Available from: 10.5205/1981-8963-v12i3a109914p651-657-2018.

[27] Agbayani CJ, Tucker JA, Nelson EL, Martinez F, Cortes H, Khoury D, et al. Immunological and psychosocial functioning in parents of children with cancer. Support Care Cancer. 2022;30(4):3379–3388. Available from: doi: 10.1007/s00520-021-06770-0 34994860 PMC9833860

