# Supplementary figures and images for: Effectiveness of multimedia education for reducing anxiety among caregivers of children and adolescents undergoing chemotherapy: Randomized controlled trial protocol
Source: PLoS One. 2023 May 9;18(5):e0285250. doi: 10.1371/journal.pone.0285250 (PMC10168554; doi:10.1371/journal.pone.0285250)

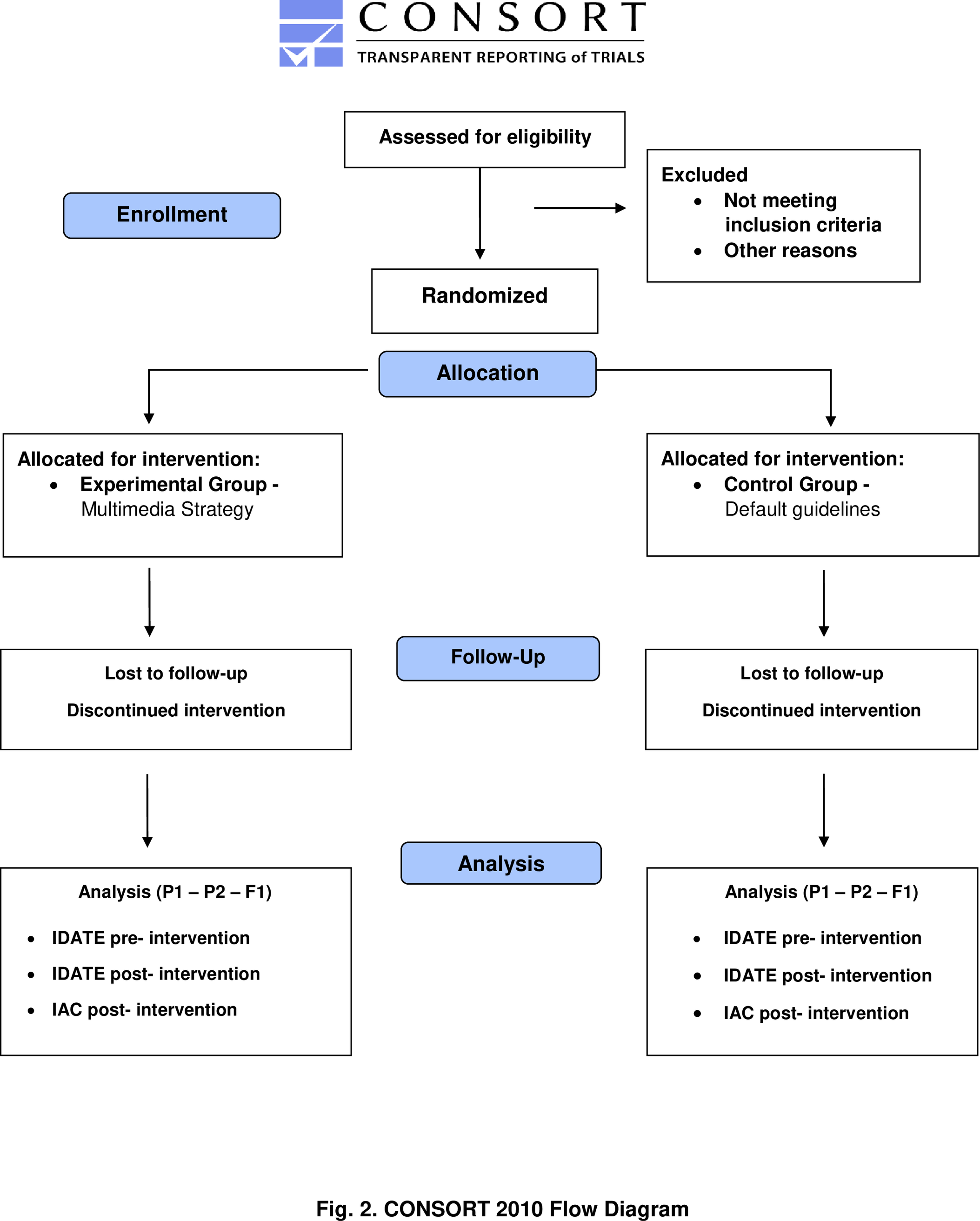

Supplement: S1 Fig 2 — (TIF) [file pone.0285250.s002.tif]
