# Supplementary material for: Effectiveness of multimedia education for reducing anxiety among caregivers of children and adolescents undergoing chemotherapy: Randomized controlled trial protocol
Source: PLoS One. 2023 May 9;18(5):e0285250. doi: 10.1371/journal.pone.0285250 (PMC10168554; doi:10.1371/journal.pone.0285250)
Supplement: S1 File — (DOC) [file pone.0285250.s003.doc]

**STANDARD GUIDELINES FOR STARTING CHEMOTHERAPY TREATMENT**

**1- Guidelines on diagnosis and treatment**

• Caregivers are oriented on what childhood cancer is, how it develops, clarifying the main doubts about the neoplasm.

• They inform about types of treatment, noting that during treatment, it may be necessary to use more than one therapeutic approach, such as surgery, radiotherapy and chemotherapy.

• There are different types of cancer and for each of them, a specific treatment is performed

• Regarding chemotherapy, the caregiver is instructed on what it is and what forms of medication that can be orally, intravenously, intramuscularly, subcutaneously and intrathecally administered. It is the main treatment for leukemias, lymphomas and most solid tumors. The caregiver is instructed on the main side effects, namely: nausea, vomiting, fatigue, hair loss and lack of appetite. Depending on the chemotherapy protocol, the child may present other side effects, such as diarrhea or constipation, myalgia and mucositis.

**2- Institution routines before chemotherapy**

• Caregivers are instructed on the importance of measuring vital signs and blood count collection before chemotherapy. The meaning of red blood cells, platelets and white blood cells is briefly explained, since they are terms that will be associated with the child's condition throughout the treatment.

**3- Risks of infection during chemotherapy treatment**

• Caregivers are advised that after chemotherapy, the child may have low defense against microorganisms (immunosuppression), facilitating the infection process; therefore, care for the child must be redoubled. It is necessary to adopt the practice of hand hygiene, wear face mask, and if the child is discharged from the hospital, avoid agglomerations.

• In case of fever of 37.8ºC, parents are advised to return to the hospital.

**4- Guidelines related to diet during chemotherapy treatment**

• Diet is essential for good prognosis, so the caregiver is informed that the child should only eat foods from known sources, well cooked, avoiding canned food, snacks or any food that is not prepared by the caregiver or provided by the hospital. It is important to have this control, because if the child has any reaction to food, the caregiver can relate it to chemotherapy. It is important to encourage the child to drink water to reduce the toxicity of chemotherapy drugs, as some chemotherapy drugs are nephrotoxic.

**5- Guidelines related to hygiene**

• The caregiver is instructed in relation to strict hygiene of the child, mainly to avoid risk of infection. Oral cavity hygiene is also encouraged, advising that some chemotherapy drugs can cause mucositis, so strict oral hygiene helps to minimize this effect.
