# Supplementary material for: Effectiveness of multimedia education for reducing anxiety among caregivers of children and adolescents undergoing chemotherapy: Randomized controlled trial protocol
Source: PLoS One. 2023 May 9;18(5):e0285250. doi: 10.1371/journal.pone.0285250 (PMC10168554; doi:10.1371/journal.pone.0285250)
Supplement: S2 File — (PDF) [file pone.0285250.s004.pdf]

BRASIL

Ir para o conteúdo [1] Ir para o menu [2] Habilitar alto contraste [3]

ReBEC
Registro Brasileiro de Ensaios Clínicos

Registro Visualizar

Procurar nos estudos

Estudo publicado

RBR-4wdm8q9 Effect of a multimedia strategy for knowledge acquisition and reduction of caregivers of children and adolescents with c...

Data de registro: 22/03/2022 (dd/mm/yyyy)

Última data de aprovação: 22/03/2022 (dd/mm/yyyy)

Tipo de estudo:

Intervenções

Título científico:

en
Multimedia Strategy for the acquisition of knowledge and reducing Anxiety of caregivers of children and adolescents in chemotherapy: randomized clinical trial

pt-br
Estratégia Multimídia para aquisição de conhecimento e redução de Ansiedade de cuidadores de crianças e adolescentes em quimioterapia: ensaio clínico randomizado

es
Multimedia Strategy for the acquisition of knowledge and reducing Anxiety of caregivers of children and adolescents in chemotherapy: randomized clinical trial

Identificação do ensaio

- **Número do UTN:** U1111-1272-6963
- **Título público:**

  en
  Effect of a multimedia strategy for knowledge acquisition and reduction of caregivers of children and adolescents with cancer

  pt-br
  Efeito de uma estratégia multimídia para aquisição de conhecimento e redução de ansiedade de cuidadores de crianças e adolescentes com câncer

- **Acrônimo científico:**
- **Acrônimo público:**
- **Identificadores secundários:**
  - **52597121.9.0000.5537**
    Orgão emissor: Plataforma Brasil
  - **5.176.784**
    Orgão emissor: Comitê de Ética em pesquisa da Universidade Federal do Rio Grande do Norte

Patrocinadores

- **Patrocinador primário:** Universidade Federal do Rio Grande do Norte
- **Patrocinador secundário:**
  - **Instituição:** Universidade Federal do Rio Grande do Norte

- **Fontes de apoio financeiro ou material:**
  - **Instituição:** Universidade Federal do Rio Grande do Norte

Condições de saúde

- **Condições de Saúde:**

en	pt-br
Multimedia; Caregiver; Neoplasms	Multimídia; Cuidador; Neoplasia

- **Descritores gerais para condições de saúde:**

en	pt-br
**I02.233.332** Health Education	**I02.233.332** Educação em Saúde

- **Descritores específicos para condições de saúde:**

en	pt-br
**J01.897.280.500.633** Multimedia	**J01.897.280.500.633** Multimídia
**SP4.046.452.698.879.165** Neoplasms	**SP4.046.452.698.879.165** Neoplasias
**M01.085** Caregivers	**M01.085** cuidadores

Intervenções

- **Intervenções:**

en	pt-br
Intervention Group: it will be composed of 26 caregivers of children/adolescents who will receive guidance with the intervention of an educational technology that is a digital animation film lasting 12 minutes and 22 seconds on the pediatric chemotherapy treatment process. Control Group: will be composed of 26 caregivers of children/adolescents who will receive only standardized verbal guidance from the health institution itself. The control and intervention groups will be randomized by simple randomization process.	Grupo Intervenção: será composto por 26 cuidadores de crianças/adolescentes que receberão as orientações com a intervenção de uma tecnologia educativa que é um filme de animação digital com duração de 12 minutos e 22 segundos sobre o processo de tratamento quimioterápico pediátrico. Grupo Controle: será composto por 26 cuidadores de crianças/adolescentes que vão receber apenas orientações verbais padronizadas da própria instituição de saúde. O grupo controle e intervenção serão aleatorizados por processo de randomização simples.

- **Descritores para as intervenções:**

en	pt-br
**J01.897.280** Educational Technology	**J01.897.280** Tecnologia Educacional

Recrutamento

- **Situação de recrutamento:** Recrutando

- **Países de recrutamento**
  - Brasil

- **Data prevista do primeiro recrutamento:** 24/01/2022 (dd/mm/yyyy)

Tamanho da amostra alvo:	Gênero para inclusão:	Idade mínima para inclusão:	Idade máxima para inclusão:
52	-	0	0

- **Critérios de inclusão:**

en
The inclusion criteria of the research are caregivers of both sexes, aged 18 years or over and who is the primary caregiver of the child or adolescent with cancer who will start chemotherapy

pt-br
Os critérios de inclusão da pesquisa são: cuidadores de ambos os sexos, com idade igual ou maior que 18 anos e que seja o cuidador principal da criança ou adolescente com câncer que irá iniciar tratamento quimioterápico

- **Critérios de exclusão:**

en
Exclusion criteria are caregivers of children and adolescents with cancer who have a disorder that makes them unable to understand and participate in the research; caregivers of children and adolescents who are starting chemotherapy treatment with disease recurrence.

pt-br
Os critérios de exclusão são cuidadores de crianças e adolescentes com câncer que apresentam algum distúrbio que incapacite de compreender e participar da pesquisa; cuidadores de crianças e adolescentes que estão iniciando tratamento quimioterápico com recidiva da doença.

Tipo de estudo

- **Desenho de estudo:**

en

Programa de acesso expandido	Enfoque do estudo	Desenho da intervenção	Número de braços	Tipo de mascaramento	Tipo de alocação	Fase do estudo
	Outro	Paralelo	2	Unicego	Randomizado controlado	N/A

Desfechos

- **Desfechos primários:**

en
- Reduction of anxiety after using the multimedia strategy, measured through the State-Trait Anxiety Inventory (STAI): >42 tends to anxiety and <38 tends to depression

pt-br
- Redução da ansiedade após uso da estratégia multimídia, medida através do Inventário de ansiedade traço-estado (IDATE): >42 tende a ansiedade e <38 tende a depressão

- **Desfechos secundários:**

en
They are not provided with secondary guarantees.

pt-br
Não são esperados desfechos secundários.

Contatos

- **Contatos para questões públicas**
  - **Nome completo:** Daniele Vieira Dantas
  -
    - **Endereço:** Petra Kelly, 61. Nova Parnamirim
    - **Cidade:** Natal / Brazil
    - **CEP:** 59152-330
  - **Fone:** +5581999367260
  - **Email:** daniele00@hotmail.com
  - **Afiliação:** Universidade Federal do Rio Grande do Norte

- **Contatos para questões científicas**
  - **Nome completo:** Daniele Vieira Dantas
  -
    - **Endereço:** Petra Kelly, 61. Nova Parnamirim
    - **Cidade:** Natal / Brazil
    - **CEP:** 59152-330
  - **Fone:** +5581999367260
  - **Email:** daniele00@hotmail.com
  - **Afiliação:** Universidade Federal do Rio Grande do Norte

- **Contatos para informação sobre os centros de pesquisa**
  - **Nome completo:** Daniele Vieira Dantas
  -
    - **Endereço:** Petra Kelly, 61. Nova Parnamirim
    - **Cidade:** Natal / Brazil
    - **CEP:** 59152-330
  - **Fone:** +5581999367260
  - **Email:** daniele00@hotmail.com
  - **Afiliação:** Universidade Federal do Rio Grande do Norte

Links adicionais:

- Download no formato ICTRP

Total de Ensaios Clínicos 11617.
Existem 5517 ensaios clínicos registrados.
Existem 3181 ensaios clínicos recrutando.
Existem 193 ensaios clínicos em análise.
Existem 4311 ensaios clínicos em rascunho.

cadastre um novo usuário
notícias
sobre

ajuda
contato
equipe
links úteis
glossário
