# Supplementary material for: Effectiveness of multimedia education for reducing anxiety among caregivers of children and adolescents undergoing chemotherapy: Randomized controlled trial protocol
Source: PLoS One. 2023 May 9;18(5):e0285250. doi: 10.1371/journal.pone.0285250 (PMC10168554; doi:10.1371/journal.pone.0285250)
Supplement: S3 File — (PDF) [file pone.0285250.s005.pdf]

# OPINION CONSUBSTANTIATED FROM CEP

**AMENDMENT DATA**

**Research Title:** MULTIMEDIA STRATEGY FOR KNOWLEDGE ACQUISITION AND REDUCTION OF ANXIETY AMONG CAREGIVERS OF CHILDREN AND ADOLESCENTS UNDERGOING CHEMOTHERAPY: RANDOMIZED CLINICAL TRIAL

**Researcher:** DANIELE VIEIRA DANTAS

**Thematic Area:**

**Version:** 3

**CAAE:** 52597121.9.0000.5537

**Proposing Institution:** Graduate Program in Nursing

**Main Sponsor:** Own Funding

**OPINION DATA**

**Opinion Number:** 5.268.320

**Project presentation:**

This research project is associated with the Graduate Program in Nursing, where 52 participants (caregivers) will be enrolled. An intervention study will be carried out, a randomized controlled trial (RCT), which will follow standards of the Consolidated Standards of Reporting Trials - CONSORT 2010 (SCHULZ; ALTMAN; MOHER, 2010), being registered on the Brazilian Registry of Clinical Trials (ReBEC) virtual platform. The research will be carried out at the 'Varela Santiago' Children's Hospital, located in the city of Natal, state of Rio Grande do Norte, Brazil. Childhood cancer affects individuals from zero to nineteen years of age, with Leukemia, Central Nervous System tumors and Lymphomas being the most prevalent types of cancer in this population. Regarding treatment, chemotherapy still remains the essential therapy to achieve cure, whether performed alone or associated with surgery and radiotherapy. Chemotherapy affects the lives not only of children and adolescents, but also of their caregivers, who usually make changes to their routine to adapt them to the new reality. Thus, health professionals, especially nurses, should be involved with the health education process, seeking educational strategies that facilitate the caregiver's understanding of guidelines and contributing to increasing knowledge and reducing the anxiety involved with the start of treatment.

**Research Purpose:**
To evaluate the effect of a multimedia strategy compared to standard guidelines for acquiring knowledge and reducing anxiety among caregivers of children and adolescents undergoing chemotherapy.

**Assessment of Risks and Benefits:**
Risks can be considered minimal.

There are direct benefits, as the study brings benefits to caregivers, children/adolescents and institution.

**Comments and Considerations on the Research:**
At this point, an amendment was presented, where the first nine participants will be used as a pilot group, evaluating IAC, IDATE instruments and the digital animation film, and data will be used to improve the techniques and stages.
Soon after, if there are discrepancies or need for adjustments to the study, information will be discarded. If there are no changes, data will be validated and included in the survey.

**Considerations on Mandatory Submission Terms:**
The amendment letter was presented, requesting the modification and comparing it with the approved project version and with the modification.

**Recommendations:**
It is the responsibility of the researcher to send the partial and final reports of the research. See models in <www.cep.propesq.ufrn.br>.
Any change in the approved protocol must first be requested through amendment, via Plataforma Brasil. See manuals at <www.cep.propesq.ufrn.br>.

**Conclusions or Pending concerns and List of Inadequacies:**
In this amendment, it was requested to increase the number of participants that will be used for the pilot trial. It is understood that the total number of participants will not change, and the objectives have not been modified. Thus, after the ethical review, we concluded that they were properly repaired.
This adequacy places the protocol within the basic precepts of ethics in research involving human beings, and is therefore approved.

GUIDELINES FOR CONDUCTING RESEARCH DURING THE SARS-COV2 PANDEMIC

Conep, the National Research Ethics Commission, through the GUIDELINES FOR CONDUCTING RESEARCH AND CEP ACTIVITY DURING THE PANDEMIC CAUSED BY THE
SARS-COV-2 CORONAVIRUS (COVID-19) document of May 9, 2020, on page 02, guides that:

**Endereço:** Rua das Artes, s/n. Campus Central UFRN.
**Bairro:** Lagoa Nova **CEP:** 59.075-000
**UF:** RN **Município:** NATAL
**Telefone:** (84)3215-3135 **Fax:** (84)99193-6266 **E-mail:** cepufrn@reitoria.ufrn.br

"3.1. It is advisable the adoption of measures for the prevention and management of all research activities, ensuring primary health actions, minimizing losses and potential risks, in addition to providing care and preserving the integrity and assistance of participants and the research team.

3.2. In compliance with the operational difficulties arising from all measures imposed by the SARS-CoV-2 (Covid-19) pandemic, it is necessary to ensure the best interest of research participants, maintaining them informed about changes to the research protocol that may affect them, especially if there is adjustment in the study conduction, schedule or work plan.

3.3. As a result, while the state of public health emergency resulting from Covid-19 lasts, it is recommended that CEPs and the entire scientific community adopt, for the conduction of research protocols involving human beings, the Conep guidelines, observing, and, where applicable, the guidelines adopted by the National Health Surveillance Agency (Anvisa).
(...)
3.6. If suspension, interruption or cancellation of the research is necessary due to the unforeseeable risks to research participants, for direct or indirect causes, it will be up to the researchers to submit a notification for consideration by the CEP/CONEP System."

It is worth mentioning that, in view of government and World Health Organization recommendations, UFRN, through Ordinance No. 452/2020-R, of March 17, 2020, in Art. 2, "authorizes extension and research activities that, by their nature, do not imply agglomeration of people, being up to researchers to evaluate the conditions for compliance with the recommendations of health authorities."

Finally, actions aimed at safeguarding those involved, participants and researchers in research activities are recommended, such as, for example, the application of online instruments (questionnaires, interviews, among others) and other measures that are considered necessary. Any doubts can be directed to our communication channels: mobile number (84) 9.9193-6266, Email address: cepufrn@reitoria.ufrn.br or contact form on the website <www.cep.ufrn.br>.

**Endereço:** Rua das Artes, s/n. Campus Central UFRN.
**Bairro:** Lagoa Nova **CEP:** 59.075-000
**UF:** RN **Município:** NATAL
**Telefone:** (84)3215-3135 **Fax:** (84)99193-6266 **E-mail:** cepufrn@reitoria.ufrn.br

**Final Considerations at the discretion of CEP:**

In accordance with Resolution 466/12 (or Resolution 510/16) of the National Health Council - CNS and Operational Manual for Ethics Committees - CONEP, the principal researcher is responsible for:

1. Elaborating the Free and Informed Consent Form - FICF in two copies, initialed on all its pages and signed, at the end, by the person invited to participate in the research or by his/her legal representative, as well as by the principal researcher, or by the person (s) delegated by him/her, and the signature pages must be on the same sheet (Res. 466/12 - CNS, item IV.5d);
2. Developing the project as outlined (Res. 466/12 - CNS, item XI.2c);
3. Submitting to the CEP any amendments or extensions with justification (Operational Manual for Ethics Committees - CONEP, Brasília - 2007, p. 41);
4. Discontinuing the study only after analysis and manifestation by the CEP/CONEP/CNS/MS System that approved it, of reasons for discontinuation, except in cases of justified urgency on the benefit of its participants (Res. 446/12 - CNS, item III.2u);
5. Preparing and presenting partial and final reports (Res. 446/12 - CNS, item XI.2d);
6. Keeping research data in a physical or digital file under his/her custody and responsibility for a period of 5 years after the end of the research (Res. 446/12 - CNS, item XI.2f);
7. Forwarding the research results for publication with due credit to associated researchers and to the technical personnel that took part of the project (Res. 446/12 - CNS, item XI.2g) and,
8. Justifying before CEP or CONEP, the interruption of the project or non-publication of results (Res. 446/12 - CNS, item XI.2h).

**Endereço:** Rua das Artes, s/n. Campus Central UFRN.
**Bairro:** Lagoa Nova **CEP:** 59.075-000
**UF:** RN **Município:** NATAL
**Telefone:** (84)3215-3135 **Fax:** (84)99193-6266 **E-mail:** cepufrn@reitoria.ufrn.br

**This opinion was prepared based on the documents listed below:**

| Type of document | File | Post | Author | Situation |
|---|---|---|---|---|
| Basic Project Information | PB_INFORMAÇAO BASICA1877733_E1.pdf | 12/20/2021 13:33:36 | | Accepted |
| Detailed project / Brochure Investigator | Projeto_Emenda_Modificado.doc | 12/20/2021 13:30:03 | DANIELE VIEIRA DANTAS | Accepted |
| Others | Carta_de_Emenda.pdf | 12/20/2021 13:28:21 | DANIELE VIEIRA DANTAS | Accepted |
| Others | Projeto_brochura_Modificado.doc | 11/21/2021 13:14:23 | DANIELE VIEIRA DANTAS | Accepted |
| Others | Carta_respostas_pendncias_pdf2.pdf | 11/21/2021 13:12:44 | DANIELE VIEIRA DANTAS | Accepted |
| Detailed project / Brochure Investigator | Projeto_CEP_s.doc | 09/20/2021 18:14:22 | DANIELE VIEIRA DANTAS | Accepted |
| Others | Termo_Autoriza_sem_assinatura.doc | 09/20/2021 17:58:37 | DANIELE VIEIRA DANTAS | Accepted |
| | | | | |
| Declaration of Institution and Infrastructure | carta_anuencia_sem_assinatura.doc | 09/20/2021 17:57:59 | DANIELE VIEIRA DANTAS | Accepted |
| Cover sheet | folhaDeRosto.pdf | 09/02/2021 07:07:18 | DANIELE VIEIRA DANTAS | Accepted |
| Declaration of researchers | Folha_de_identificac807a771o_do_pesquisador.pdf | 09/02/2021 06:59:45 | DANIELE VIEIRA DANTAS | Accepted |
| Others | Declara_compromisso.pdf | 09/02/2021 06:59:21 | DANIELE VIEIRA DANTAS | Accepted |
| FICF / Terms of assent / Justification of Absence | FICF.pdf | 09/02/2021 06:54:11 | DANIELE VIEIRA DANTAS | Accepted |
| Declaration of Institution and Infrastructure | carta_anuencia_pdf.pdf | 09/02/2021 06:53:43 | DANIELE VIEIRA DANTAS | Accepted |

| | | | | |
|---|---|---|---|---|
| | Termo_confidencialidade.pdf | 09/02/2021 06:53:22 | DANIELE VIEIRA DANTAS | Accepted |
| Others | Autoriza_estrategia_pdf.pdf | 09/02/2021 06:52:14 | DANIELE VIEIRA DANTAS | Accepted |
| Budget | orcamento.doc | 09/02/2021 06:22:42 | DANIELE VIEIRA DANTAS | A Accepted |
| Schedule | cronograma.doc | 09/02/2021 06:21:28 | DANIELE VIEIRA DANTAS | Accepted |

**Opinion Status:**

Approved

**Requires CONEP Appraisal:**

No

NATAL, March 02, 2022

Paula Fernanda B. B. dos Santos

Paula Fernanda Brandão B. dos Santos
Docente UFRN
Mat. 1529790

**Signed by:**
**PAULA FERNANDA BRANDÃO BATISTA DOS SANTOS**
**(Coordinator)**

**Endereço:** Rua das Artes, s/n. Campus Central UFRN.
**Bairro:** Lagoa Nova **CEP:** 59.075-000
**UF:** RN **Município:** NATAL
**Telefone:** (84)3215-3135 **Fax:** (84)99193-6266 **E-mail:** cepufrn@reitoria.ufrn.br
