# Supplementary material for: Effectiveness of multimedia education for reducing anxiety among caregivers of children and adolescents undergoing chemotherapy: Randomized controlled trial protocol
Source: PLoS One. 2023 May 9;18(5):e0285250. doi: 10.1371/journal.pone.0285250 (PMC10168554; doi:10.1371/journal.pone.0285250)
Supplement: S4 File — (DOC) [file pone.0285250.s006.doc]

**GRAPHIC OUTLINE FOR THE PROTOCOL**

Child/adolescent is admitted for chemotherapy treatment

Research Team:

- Approach the child-adolescent caregiver

- Check Eligibility Criteria

Caregiver does not meet Eligibility Criteria

Caregiver meets Eligibility Criteria

**Research Team:**

- Clarification of the research, risks and benefits

- Application of the TCLE

Disregard from Research

**Caregiver directed to:**

Group control

Experimental Group

**- P1**: Apply STAI

**Duration:** average of 10 minutes

**P2: Main Researcher:**

Performs the intervention using verbal guidelines (institution's standard guidelines)

**Duration**: average of 20 minutes

**F1:** After 30 minutes of the intervention, apply the STAI + IAC.

**Duration**: average of 30 minutes.

**Duration time between P1 and F1:** 90 minutes.

**P1:** Apply STAI

**Duration:** average of 10 minutes

**P2: Main Researcher:**

Performs the intervention using an educational technology - digital animation film about the chemotherapy process to assist in the guidelines.

**Duration**: average of 20 minute

**F1:** After 30 minutes of the intervention, apply the STAI + IAC.

**Duration**: average of 30 minutes.

**Duration time between P1 and F1:** 90 minutes.

**Subtitle:**

**P1:** Day 1 - start of chemotherapy

**P2:** Intervention Period

**F1:** Assessment 30 minutes after intervention
