# Supplementary material for: Effectiveness of multimedia education for reducing anxiety among caregivers of children and adolescents undergoing chemotherapy: Randomized controlled trial protocol
Source: PLoS One. 2023 May 9;18(5):e0285250. doi: 10.1371/journal.pone.0285250 (PMC10168554; doi:10.1371/journal.pone.0285250)
Supplement: S5 File — (DOC) [file pone.0285250.s007.doc]

**
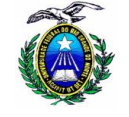
**

**FEDERAL UNIVERSITY OF RIO GRANDE DO NORTE**

**HEALTH SCIENCES CENTER**

**STRICT SENSU GRADUATE PROGRAM IN NURSING**

**MULTIMEDIA STRATEGY FOR THE ACQUISITION OF KNOWLEDGE AND REDUCING ANXIETY OF CAREGIVERS OF CHILDREN AND ADOLESCENTS IN CHEMOTHERAPY: RANDOMIZED CLINICAL TRIAL**

**NATAL/RN**

**2021**

**ABSTRACT**

The study aims to evaluate the effect of a multimedia strategy compared to standard guidelines for knowledge acquisition and anxiety reduction in caregivers of children and adolescents undergoing chemotherapy. This is a randomized and controlled clinical trial, which will be carried out in a Philanthropic Hospital, a reference in the care of children and adolescents with cancer, in Natal-RN. The Experimental Group will receive the institution's standard guidelines associated with a multimedia strategy on the chemotherapy treatment process - Digital animation film lasting 12 minutes and 22 seconds, while the Control Group will only receive the verbal guidelines that are provided at the institution. The data collection instrument used will be the State-Trait Anxiety Inventory (STAI) and a Knowledge Assessment Instrument (IAC). For data collection, the study will have the main researcher and two other researchers to apply the instruments. Researcher 1 will apply the instrument to assess anxiety in all participants before chemotherapy treatment, the main researcher will perform interventions in both groups, and researcher 2 after the interventions, without knowing which participant belongs to which group, will apply the anxiety instrument and the instrument to assess the knowledge acquired in both groups. Regarding data analysis, descriptive and inferential statistics will be performed.

**Keywords**: Multimedia; Caregiver; Cancer; Child; Adolescent.

**INTRODUCTION**

Cancer is considered a chronic disease and represents a public health problem in Brazil, being the leading cause of death from disease among children and adolescents aged one to ten years. Because its development is marked by uncertain care and undisclosed risk factors, it becomes a challenge for the scientific community. Being leukemias, tumors of the Central Nervous System (CNS) and lymphomas the main types of malignant neoplasms in childhood^[1]^.

Regarding treatment, chemotherapy is still the basis of therapy and can be used in association with other methods, such as surgery and radiotherapy. Due to the long periods of hospitalization, both for the child and for the family and/or main caregiver, there is a break in the routine of their activities – an illness process for all involved. In this way, the caregiver of the child/adolescent goes through a process of change and coping that requires support to perform the task of caring^[2]^.

Through this prism, chemotherapy treatment causes some side effects, which are surrounded by meanings of fear, stigma and anxiety on the part of parents, family members or caregivers responsible for children and adolescents, mainly because they do not know the therapy, its effects and the purpose of the treatment^[3]^.

It is noteworthy that this lack of knowledge on the part of caregivers causes, mainly, anxiety, which is one of the most common symptoms of those who experience the chemotherapy process. Health education is one of the ways associated with reducing anxiety, due to the clarification of doubts and necessary guidelines for care with chemotherapy ^[4,5]^.

It is necessary to take into account that the caregiver may not have sufficient training to understand technical terms, and it is necessary to facilitate communication, using language accessible to all, which is enlightening, simple and objective. And that allows, based on doubts, to guide the caregiver ^[4]^.

In this context, the use of multimedia strategies arises, which represent a set of resources that stimulate all the senses, the most common being vision and hearing. They can be considered as an Educational Technology when they are developed for the teaching-learning process^[6]^.

It should be noted that there are still few interventions using educational technologies for the child's caregiver, requiring the development of resources that can help the parents and family who care for the child with cancer^[7]^.

Taking into account multimedia strategies and the need to facilitate the educational process for caregivers of children and adolescents undergoing chemotherapy, the study is based on the hypothesis that the use of a multimedia strategy can be effective in guiding caregivers and consequently reducing the level of anxiety when compared with the institution's verbal guidelines.

**OBJECTIVE**

To evaluate the effect of a multimedia strategy compared to standard guidelines for knowledge acquisition and anxiety reduction in caregivers of children and adolescents undergoing chemotherapy.

**MATERIALS AND METHODS**

**Trial design**

An intervention study will be carried out, a controlled randomized clinical trial (RCT) that will follow the standards of the Consolidated Standards of Reporting Trials - CONSORT 2010^[8]^, being registered on the Brazilian Registry of Clinical Trials (ReBEC) virtual platform .

**Study Scenario**

Recruitment and intervention will be carried out at the largest philanthropic pediatric hospital that is a reference in the care of children and adolescents with cancer, located in the city of Natal, state of Rio Grande do Norte, Brazil.

**Study Population**

Caregivers of children and adolescents diagnosed with some type of cancer who will start chemotherapy treatment.

**Sample size**

The sample is probabilistic and simple random. Sample size calculation was performed using the G Power software, version 3.1.9.2 (available at: http://www.gpower.hhu.de/). The calculation was performed considering the study by Bernardi et al (2019)^[9]^ that evaluated the IDATE score in caregivers of children and adolescents with cancer. Thus, considering the Cohen effect size of 0.80, test power of 0.80 and significance level of 5% (p-value < 0.05), the study had 26 participants in the Control Group (CG) and 26 participants in the Experimental Group (EG), totaling a sample of 52 study participants.

**Eligibility Criteria**

Caregivers of both sexes, aged 18 years or older, who are the main caregivers of the children or adolescent with cancer who will start treatment chemotherapy in the ward will be included.

Participants who present the following conditions will be excluded from the study: disorder that prevents understanding and participation in the research; caregivers of children and adolescents who are starting chemotherapy treatment with disease recurrence; caregivers who have already had experience in caring for patients undergoing chemotherapy, individuals with visual or hearing deficits; caregivers of children and adolescents undergoing outpatient chemotherapy.

**Recruitment**

Caregivers will be recruited between January and December 2022, when the child/adolescent is admitted to the pediatric oncology sector of the hospital where the study will be carried out. Caregivers will be contacted and eligibility criteria for research participation will be applied.

Subsequently, the study, interventions and instruments used for data collection will be presented to recruited participants and any doubts that may arise about the study will be addressed. Thus, participants who agree to participate in the study will receive a consent form for reading and signing. The study explanation and the free and informed consent form will be provided by the main researcher. It is noteworthy that participants will have their personal data protected.

**Allocation**

The randomization of participants will be carried out through the www.randomizer.org website, which will previously select participants for each group (CG and EG) by simple randomness and without the influence of researchers. The site generates a list with the sequence of participants equally divided into the two intervention groups. It is noteworthy that the allocation of participants will be carried out by a researcher who is not involved in the intervention. Additionally, the allocation will be hidden.

It is noteworthy that randomization will avoid participant selection bias and will allow groups to be compared in terms of their known and unknown risk factors.

**Intervention**

The intervention that will be used in EG is the application of a multimedia strategy associated with the Institution's standard guidelines. The multimedia strategy is the exhibition of a digital animation film with duration of 12 minutes and 22 seconds addressing the pediatric chemotherapy treatment process.

The film is an educational technology developed by Pinheiro et al (2020)^[10]^, during the master's degree of the main author, using as reference a manual of a service of Hematology and Oncology of the University Hospital of Santa Maria (HUSM), specialized in the treatment of childhood cancer in partnership with students and professors of the Design, Digital Games and Journalism courses at the Franciscan University (UFN). It was validated by experts in the field and by the target audience and authorized to carry out the study, but it is not available for free access.

The digitally animated film will be shown in a quiet room, before starting chemotherapy. The caregiver will be provided with a headset and a tablet to watch the movie.

The digital animation film used in the study generally addresses the concept of chemotherapy, chemotherapy administration routes, most frequent side effects and general care during treatment. The film emphasizes health education about chemotherapy for the caregiver.

GC will receive instructions verbally. The standard verbal instructions are provided as supplementary material.

The intervention period will be carried out in two days consisting of three stages: the first stage is performed on the first day of contact after admission of the child/adolescent to the chemotherapy unit with the caregiver who, after accepting and signing the informed consent form, will be included in the search.

The second stage is performed on the second day of contact, which will be on the child's first day of chemotherapy, and before starting the procedure, the main researcher will apply the instrument to assess the level of anxiety (State Trait Anxiety Inventory - STAI) of caregivers in both groups (CG and EG).

Subsequently, the intervention will be carried out according to the groups formed, in which the principal investigator will carry out health education using the multimedia strategy associated with the standard guidelines in the EG and standard guidelines in the CG.

The third stage will be carried out on the second day 30 minutes after the end of interventions in which a second researcher, trained to apply the STAI and KAI and who has no understanding of patient allocation, will apply these instruments to participants of both groups, CG and EG.

A pilot test will be carried out with the first nine participants, which will serve for research analysis in order to adapt instruments and the methodological approach as necessary to meet the objectives, for better adherence to the intervention protocol.

**Data Collection Instrument**

Two instruments will be used for data collection:

1) State Trait Anxiety Inventory (STAI) ^[11]^, an instrument that assesses trait and state anxiety, the first relates to the caregiver’s usual anxiety and the second concerns transient anxiety related to some stressful event. Thus, the aim was to obtain the level of anxiety of participants before and after intervention, comparing these values ​​to evaluate the management of anxiety by intervention, as well as comparing values between groups, evaluating the best management.

2) Knowledge Assessment Instrument (KAI), adapted from the study by Oliveira, Souza and Pellanda (2016) ^[12]^, which has the aim of assessing the effectiveness of the multimedia strategy used in the acquisition of knowledge by participants.

This instrument has a semi-structured questionnaire composed of three stages: the first consists of sociodemographic data of participants; the second contains information about previous guidelines by other professionals and the participant's opinion about the intervention performed in order to report whether the intervention was able to resolve doubts about the chemotherapy procedure. The third stage consists of nine questions about the chemotherapy process, to quantify the knowledge that participants obtained on the subject after intervention. The STAI and KAI are validated and reliable instruments used in studies with adults and caregivers.

The Knowledge Assessment Instrument (IAC) is a form prepared by the main researcher based on the questionnaire used in the study by Oliveira, Souza and Pellanda (2016) in portuguese^[12]^.

**Data collection and management**

Researchers will ensure that the anonymity of participants is protected and data collected will remain confidential so that their identities and any type of identifying information are protected. Two copies of the informed consent form will be provided, both signed by participants, researchers and guardians.

After signing the consent form, the patient may terminate his/her participation in the study at any time, if desired, without any consequences related to his/her treatment or follow-up at the institution. The results of this research can be presented at meetings or publications; however, the identity of participants will not be revealed.

**Data analysis**

Data collected in this research will be stored and processed in computerized database using the Microsoft Office Excel 2010 and Statistical Package for the Social Sciences (SPSS) version 20.0 software. Descriptive and inferential statistics of collected variables will be performed, and data will be presented through tables, charts and figures.

Statistical analysis of comparison and correlation of data obtained will be performed. The Kolmogorov-Smirnov test will be performed to analyze sample normality. Likewise, Pearson or Spearman tests will be used for correlational analyses. Categorical variables will be analyzed using the chi-square test or Fisher's exact test. To compare the KAI and STAI assessment with the intervention and control groups, Student's t-test will be applied. Comparing the IAC and IDATE classification with the group of intervention and control, the chi-square test (χ²) will be applied. Being adopted throughout the study a significance level of 5% at which p=0.05.

**Graphic outline for the protocol**


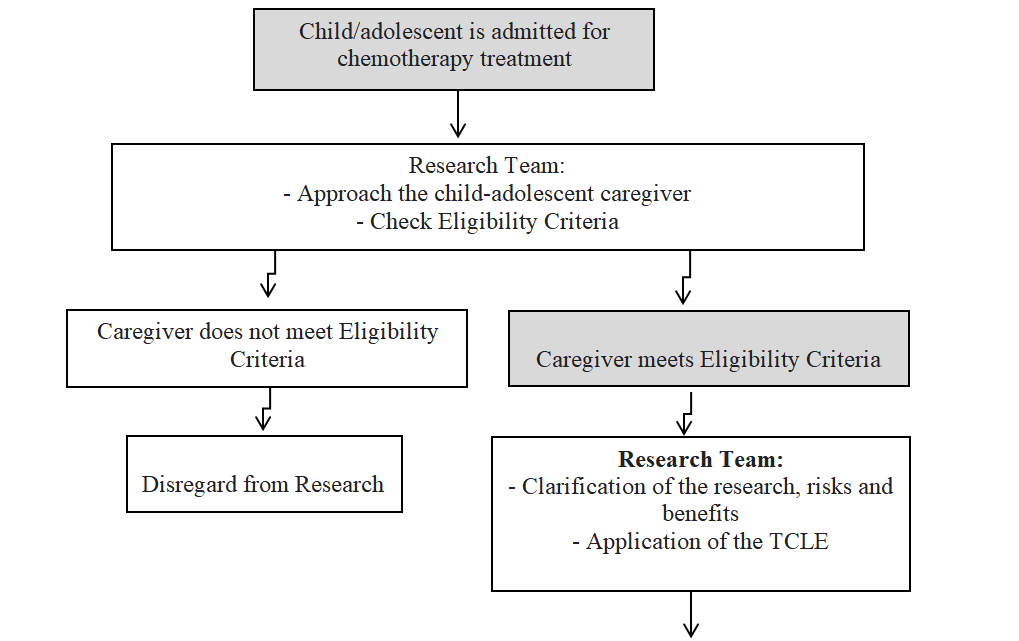


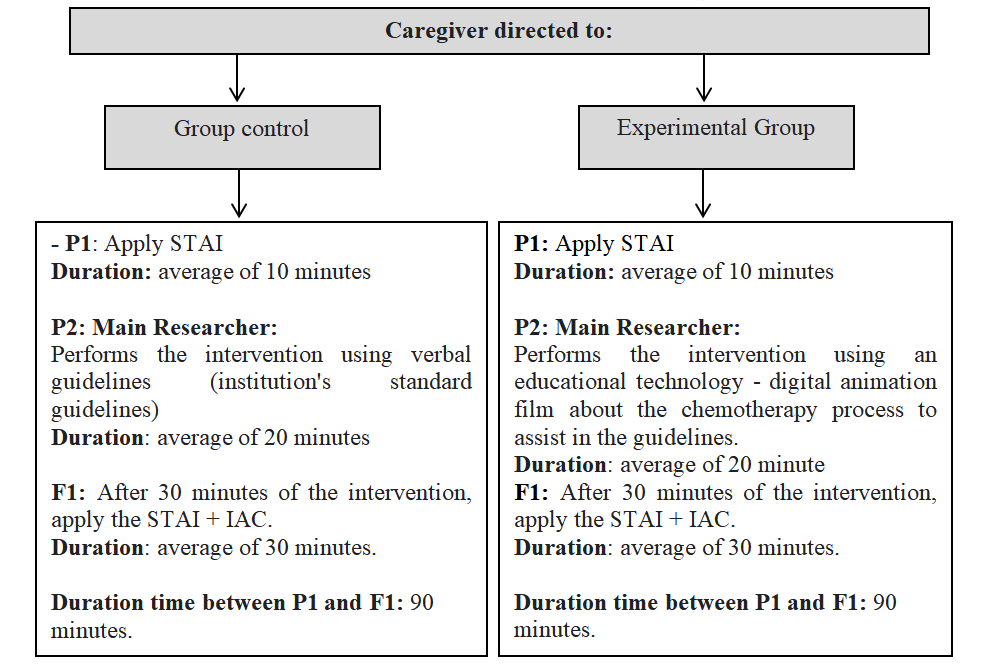


**Ethical considerations**

The present study was analyzed and approved by the Research Ethics Committee of the Federal University of Rio Grande do Norte – UFRN –CAAE— 52597121.9.0000.5537 (03/02/2022) – and CEP 5.176.784 (03/02/2022). This study was also registered in the Brazilian Registry of Clinical Trials – REBEC–RBR-4wdm8q9 as a clinical trial (03/22/2022).

The participation of patients will be carried out on a voluntary, non-profit basis and all stages, objectives, risks and benefits of the research will be explained. Participants can drop out the study at any stage without prejudice to treatment, without suffering penalties or judgments of any kind.

**Subtitle: P1:** Day 1 - start of chemotherapy**; P2:** Intervention Period **F1:** Assessment 30 minutes after intervention

**Ethical considerations**

The research project will be submitted to the approval of the Research Ethics Committee of the Federal University of Rio Grande do Norte (CEP UFRN) for appreciation and approval.

The participation of patients will be carried out on a voluntary basis, without profit and will occur by reading and signing the Term of Free and Informed Consent-TCLE, which demonstrates that the individual accepts to participate in the research, explaining all the steps, objectives , risks and benefits of the research. It is possible for participants to withdraw at any stage of the research without prejudice to the treatment, without suffering penalties or judgments of any kind.

**Risk analysis**

Regarding risks, caregivers may experience hearing discomfort due to the volume, but this will be mitigated by adjusting the volume by the researcher during the intervention, adapting to the caregiver's preference. With regard to the risk of infection due to the use of equipment used during the intervention, they will be minimized by not sharing them until everyone is presented with 70% ethyl alcohol, in addition to hand hygiene of the investigators involved before and after each procedure.

All researchers will use Personal protective equipment such as a surgical mask and lab coat for contact with participants, in addition to sanitizing their hands before and after contact with each participant. Data collection will not be carried out in the presence of patients with flu-like symptoms suspected of COVID-19. Also, a favorable point for developing the research and reducing the risk of infection by Covid-19 is the fact that the researchers involved in the research are already vaccinated, as well as a large part of the population will be vaccinated when the research is carried out in the field.

As for the confidentiality of the data, these will only be used for scientific purposes, in which the names will be acquired in secrecy and anonymity in the database to guarantee the confidentiality and non-exposure of the participants.

**Benefit Analysis**

The study brings benefits to the caregiver, child/adolescent and institution. For the caregiver, it will be possible to strengthen knowledge as well as provide new learning with the use of the multimedia strategy associated with the standard guidelines that are made available by the team. In addition, it will be possible to verify whether the multimedia resource helps to increase knowledge and reduce the level of anxiety of the caregiver.

For a child/adolescent, it helps because when you have a well-oriented caregiver, the treatment process flows more effectively, providing a confrontation with the child/adolescent with support from the main caregiver, who can clarify doubts that arise about chemotherapy for the little ones.

For the institution, the benefits are related to the health education process, allowing the pediatric oncology team to use a resource that contributes to teaching in a humanized way, considering that the film was designed for the child undergoing chemotherapy treatment.

**REFERÊNCIAS**

1. Brasil. Instituto Nacional do Câncer. Estimativa 2020-Síntese de Resultados e Comentários. [internet]. 2020 [cited 2021 jul. 17]. Available from: <https://www.inca.gov.br/estimativa/sintese-de-resultados-e-comentarios>.
2. Oliveira JS, Cunha DO, Santos SC, Morais RL. Repercussions on the lives of caregivers of children and adolescents with oncological disease. Cogitare Enferm. 2018; 23 (2):e51589. Available from:: [http://docs.bvsalud.org/biblioref/2018/06/904957/51589-233962-1-pb.pd](http://docs.bvsalud.org/biblioref/2018/06/904957/51589-233962-1-pb.pdf)f. Acesso em: 10 jun. 2021.
3. Rodrigues JRG, Siqueira Junior AC, Siqueira FPC. Nursing consultation in pediatric oncology: a tool for empowering parents. J. res.: fundam. care. 2020; 12(1): 211-22, 2020. Available from: http://seer.unirio.br/index.php/cuidadofundamental/article/view/7569/pdf .
4. Hamdan AB, Ballourah W, Elghazaly A, Javison S, Alshammary S, Erlandez R, et al. The Effect of Video-Assisted Education Prior Intrathecal Chemotherapy on Anxiety and Knowledge Enhancemen. J Cancer Educ. 2020;20(1):65-70. Available from: <https://doi.org/10.1007/s13187-020-01787-1>
5. Garcia S. The Effects of Education on Anxiety Levels in Patients Receiving Chemotherapy for the First Time: An Integrative Review. Journal Club Article, v. 18, n.5, p.516-521, 2014. Disponível em: https://pubmed.ncbi.nlm.nih.gov/25164233/. Acesso em: 20 jun. 2021.
6. Sun V, Raz DJ, Ruel N, Chang W, Erhunmwunsee L, Reckamp K, et al. A Multimedia Self-Management Intervention to Prepare Cancer Patients and Family Caregivers for Lung Surgery and Post-Operative Recovery. Clinical Lung Cancer. 2017;18(3):151-159. Available from: <https://www.ncbi.nlm.nih.gov/pmc/articles/PMC5413411>.
7. Nova F, Allenidekania A, Agustini N. The effect of multimedia-based nutrition education on parents’ knowledge and body weight change in leukemia children. Enferm Clin. 2019;29(5):230-3. Available from:<https://doi.org/10.1016/j.enfcli.2019.04.027>
8. Schulz KF, Altman DG, Moher D; CONSORT Group. CONSORT 2010 statement: updated guidelines for reporting parallel group randomised trials. BMJ. 2010;340:c332. Available from: <https://bmcmedicine.biomedcentral.com/articles/10.1186/1741-7015-8-18>
9. Bernardi MLD, Amorim MHC, Salaroli LB, Zandonade E. Effects of Hatha Yoga on caregivers of children and adolescents with cancer: a randomized controlled trial. Escola Anna Nery. 2019; 24(1):e2019013.  <https://doi.org/10.1590/2177-9465-EAN-2019-0133>.
10. Pinheiro M, Vieira AS, Sasso T, Oliveira MF, Abaid JLW, Filippin NT. We are your friends: a film of digital animation for children in chemotherapy treatment. Research, Society and Development. 2020; 9(12):2-15. Available from: <http://dx.doi.org/10.33448/rsd-v9i12.11253>.
11. Biaggio AMB, Natalício L, Spielberger CD. Development of the experimental form in Portuguese of the State-Trait Anxiety Inventory (STAI): by Spielberger. Arquivos Brasileiro de Psicologia Aplicada. 1977; 3 (29): 31-44. Available from: http://bibliotecadigital.fgv.br/ojs/index.php/abpa/article/view/17827.
12. Oliveira APA, Souza EM, Pellanda LC. Effectiveness of video resources in nursing orientation before cardiac heart surgery. Rev. Assoc. Med. Bras. 2016;62 (8):762-767. Available from: <https://www.scielo.br/j/ramb/a/ydPt75qjBn4ghypvB5STx3G/abstract/?lang=en>.
